# Clinical Manifestations in a Fatal Case of Probable *Rickettsia* and *Leptospira* Coinfection in Yucatan, Mexico

**DOI:** 10.3390/pathogens10080914

**Published:** 2021-07-21

**Authors:** Karla R. Dzul-Rosado, Maria Fidelia Cardenas-Marrufo, Cesar Lugo-Caballero, Alberto Alvarez-Baeza, Nina Mendez-Dominguez

**Affiliations:** 1 Center for Regional Research, “Dr. Hideyo Noguchi” Biomedical Unit, Mérida 97000, Mexico; karla.dzul@correo.uady.mx (K.R.D.-R.); cesar.lugo@correo.uady.mx (C.L.-C.); 2 Faculty of Medicine, Universidad Autónoma de Yucatán, Mérida 97000, Mexico; cmarrufo@correo.uady.mx; 3 School of Medicine, Universidad Marista de Mérida, Mérida 97300, Mexico; abaeza1094@gmail.com; 4 Hospital Regional de Alta Especialidad de la Península de Yucatán, Mérida 97133, Mexico

**Keywords:** coinfection, *Leptospira*, rickettsial infections, endemic diseases, hygiene, signs and symptoms

## Abstract

Clinical or serological coinfections of *Rickettsia* and *Leptospira* are uncommon but should be included in differential diagnosis when poor sanitation and cohabitation with infected animals may converge. Rickettsial and leptospiral infections have been continuously increasing throughout the past decade in Yucatan, Mexico. Leptospirosis is a zoonotic disease caused by aerobic spirochetes, while rickettsiosis is an arthropod-borne disease. In 2020, 16% of all rickettsiosis cases and 10% of leptospirosis in the country originated in Yucatan. The objective of the present case report was to document an unusual case of probable coinfection with *Rickettsia* and *Leptospira* with emphasis on clinical manifestations and the epidemiological context that may orient future multidisciplinary measures. Here, we presented the case of a 12-year-old female whose mother had recently recovered from a rickettsial infection. The patient presented with fever and developed unspecific signs and symptoms of infection; however, her condition quickly deteriorated with gastrointestinal, hepatic, renal, and neurological dysfunction. After discounting rabies and identifying infection with *Rickettsia* and *Leptospira*, antibiotic treatment was indicated, but it was too late to prevent death. Simultaneous infections of *Rickettsia* and *Leptospira* may be considered in endemic regions when environmental, epidemiological, and clinical conditions converge.

## 1. Introduction

*Rickettsia* and *Leptospira* infection incidence has been continuously increasing among the pediatric population in the past decade, particularly in disadvantaged rural areas of Yucatan, Mexico [1]. Leptospirosis is a zoonotic disease caused by aerobic spirochetes [2], while rickettsiosis is an arthropod-borne disease caused by Gram-negative bacteria [3]. In the region where the case took place, severe and fatal *Rickettsia* infections in pediatric and adult patients have been associated with ticks carried by cats and dogs [4]. Rickettsiosis in humans is characterized by clinical signs including fever, rash, and lymphadenopathy along with symptoms including malaise, myalgia accompanied by vomiting, diarrhea, and headache. Lesions from inoculation can often be found, and unusual severe manifestations can be of neurologic nature [5].

Human leptospirosis can be considered to be an invasive infectious and systemic inflammatory disease directly associated with environments with poor hygiene and direct exposure to urine from infected reservoirs carrying these pathogens in their renal tubules or indirect exposure to contaminated soil and contaminated water sources used for drinking or submersion. Additionally, mucosal/skin barriers can become an entry port and allow infection [6]. Manifestations are diverse, presenting with febrile illness, chills, headache, myalgia, abdominal pain, conjunctival suffusion, and, less often, a skin rash. Severe complications include acute renal failure and pulmonary affection such as hemorrhagic pulmonary leptospirosis, which can lead to death [7,8], and aseptic meningitis has been reported in leptospirosis among younger patients [9].

Many houses located in rural areas of Yucatan still have no access to running water. In some villages, pumps are activated once or twice a day and residents collect and keep water in containers. In even more deprived areas, rainwater is collected using wells. Cenotes, not superficial lakes, are the source of freshwater, but as cenotes are connected underwater, experts proved that the aquifer in the region is vulnerable to contamination by human activities [10]. During the rainy season, water is not easily absorbed by the rough soil, generating puddles where animals find drinking sources. Leptospirosis prevalence in humans in the region was reported to amount to 14.1% in 1984 and 14.2% in 2002; it was more prevalent in rural areas than in urban ones, with 74.3% of the reported cases occurring during the rainy season [11]. In a seroprevalence study conducted on humans in 2018 in the same area, seropositivity of 35% was reported [12].

Domestic dogs and cats in rural Yucatan are commonly not intradomicile pets; they linger most of the time in public areas and defecate freely anywhere, and no measures for remediating animal waste have been implemented. In 2011, in a reservoir seroprevalence study, the majority of dogs and cats slept outdoors, and 45.2% of the free-roaming dogs and 15.2% of the cats were found to be positive for *Leptospira* [8,13]; however, dogs may be involved in not only the transmission of *Leptospira*, but also that of *Rickettsia*, as dogs are in contact with wild animals (such as deer or wild swine), as well as with other dogs, cattle, and their human owners. It is not uncommon to find them carrying ectoparasites such as ticks which may transmit *Rickettsia*, as previously reported in the studied region [4].

In 2020, official epidemiological registries of passive surveillance reported 600 confirmed cases of *Rickettsia* infections in Mexico; 16% of them occurred in Yucatan [1]. Additionally, 87 new cases of leptospirosis were confirmed nationwide, of which 10% corresponded to the state of Yucatan; as in other areas of Latin America, underdiagnosis may be a common problem with both diseases [14]. The objective of the present case report was to document an unusual probable case of rickettsiosis and leptospirosis coinfection with emphasis on clinical manifestations and the epidemiological context that may orient future multidisciplinary preventive measures.

## 2. Case Presentation

The subject of this case was a 12-year-old female from Yucatan, Mexico. She cohabitated with her parents, an older brother, and a grandfather. The household included a backyard containing fruit trees, chickens that were bred for self-subsistence, and a latrine. Two cats and two domestic dogs had free access to the house. No running water was available indoors. Her parents reported her being vaccinated according to the basic immunization scheme, prior symptoms indicative of chickenpox at age nine, recurrent urinary tract infections, and bacterial gastroenteritis at age eleven.

The patient debuted with intermittent fever up to 39 °C for six days accompanied by incapacitating frontal headache irradiating to the occipital area accompanied by photophobia, epigastric oppressive pain, anorexia, nausea, and vomiting four to six times a day. She received medical attention at the rural medical unit and was treated ambulatorily with acetaminophen and oral rehydration. As vomiting and fever persisted, she was taken to the emergency room at the rural hospital. At admission, she was described as anxious, uncooperative, with altered speech, and she was perceived as irritable and even described as aggressive. Physical examination revealed a fever of 39.1 °C, hyporeflective isochoric pupils, and positive atypical Kernig’s and Brudzinski’s signs (consistent with meningitis). She was treated with penicillin G procaine plus metamizole and oral meclizine.

On day one of hospitalization, her blood analysis showed leukocytosis of 21,700/μL and 71% neutrophils and eosinophilia, along with total proteins of 9.4 g/dL, albumin of 5.5 g/dL, high levels of hepatic enzymes, and glucose of 198 mg/dL. She was diagnosed with gastrointestinal bacterial infection.

On day two of hospital stay, neuroinfection was suspected; the patient was transferred to the general hospital at the state capital city. Treatment was changed by adding third-generation cephalosporins, but penicillin was halted, and neuroprotection measures were implemented (analgesia, adequate sedation, and thromboembolic prevention). During her stay, she maintained a fever up to 39 °C, generalized rash with scratch marks in the back of the scalp and behind the right ear were noted, and she also had small scars consistent with flea or tick bites. Physical examination showed hepatomegaly and enlargement of lymph nodes in the retroauricular, axillary, and inguinal regions. No visible signs of mammalian bites or wounds were found or reported by the patient or her relatives, but her mother provided a personal medical report of her recently resolved rickettsial infection (a month before her daughter debuted with a fever). No other relative reported a fever or infections in the previous month. Vancomycin was added to her antimicrobial therapy at the meningeal dose. Cerebral edema was identified by tomography, and intravenous solutions were adjusted to prevent intracranial hypertension.

Rabies was the suspected diagnosis, and an observation of involved domestic dogs in search of clinical signs of rabies and behavior changes was conducted, but no suggestive manifestations were found. Additionally, domestic animals living in the village were immunized during a campaign six months before the incident. Laboratory tests for rabies according to the Mexican National Center for Disease Control and Preventive Programs were indicated for the patient, and a lumbar puncture was performed to test cerebrospinal fluid (CSF), but it tested negative.

The patient presented major neurological deterioration with loss of consciousness and generalized tonic–clonic seizures, requiring orotracheal intubation and admission to the pediatric intensive care unit where her treatment included mechanical ventilation, third-generation cephalosporins, vancomycin, and hydralazine.

After two days at the intensive care unit (day four of hospital stay), acute kidney injury (AKI) was diagnosed. With the antecedent of having contact with a confirmed case of *Rickettsia* and based on the persistent dermatologic manifestations, the patient was tested for *Rickettsia* and *Leptospira* with consideration of the type of water access, and a sample of CSF was sent for *Rickettsia* molecular and serologic diagnosis.

Serologic diagnosis of *Leptospira* spp.: Antibodies were approached using the microscopic agglutination test (MAT) [11]. MAT is considered the gold standard of reference for the diagnosis of leptospirosis and was performed using live antigens at the laboratory of the Faculty of Medicine of the Autonomous University of Yucatan under the World Health Organization’s protocols. The antigens used were from serogroups canicola, hardjo, pyrogenes, panama, pomona, tarassovi, icterohaemorrhagiae, gryppotyphosa, wolffii, autumnalis, australis, and bratislava. A result is considered positive when the serum shows at least 50% agglutination with one or more serogroups, with further titration in serial twofold dilutions starting from 1:100. The patient’s serologic test reported positive for leptospirosis, with 1:400 for *L. interrogans* serovar *australis* (applicable positive case definitions include a single MAT ≥ 1:400 in endemic regions or a single MAT ≥ 1:100 in non-endemic regions along with symptomatology for positivity) [15].

Serologic diagnosis of *Rickettsia*: In-house indirect immunofluorescence assay for IgG and IgM antibody titers against *Rickettsia* from *R. rickettsia* (SFG) and *R. typhi* (TG) of serum samples were determined [16].

Molecular diagnosis of *Rickettsia*: The CSF sample was collected, and DNA was extracted using a QIAamp DNA Blood Mini Kit (QIAGEN Valencia, CA, USA) in accordance with the manufacturer’s instructions. *Rickettsia* spp. were determined by sequencing using genus-specific primers for the rickettsial rompB and citrate synthase (gltA), with 98% homology to *Rickettsia rickettsii*. The sensitivity was between 93% (OmpA semi-nested + OmpB nested) and 100% (OmpA semi-nested + OmpB nested + gltA) [14]. The immunofluorescence reagent for *R. rickettsii* IgM 1:256 and IgG 1:512 was positive; thus, CSF was positive for *R. rickettsii*.

On day five of stay at the intensive care unit (day seven of hospital stay), vancomycin and acyclovir were suspended, and treatment with doxycycline was initiated at the conventional dosage. On day six of intensive care (day eight of hospital admission), the patient developed clinical manifestations consistent with sepsis; infection was found in cultures from the bloodstream and the urinary tract, and hemodynamic decompensation started a few days later. After ten days of hospitalization at the intensive care unit, the patient developed brain death. No other infective agents were identified during her stay. She stayed at the intensive care for 33 days until declared dead. A death review of this case by an expert committee was performed, and after the review, the direct cause of death was determined to be septic shock due to *Rickettsia* and *Leptospira*; no other comorbid condition diagnoses were included, as the patient did not have any communicable or noncommunicable diseases that could exacerbate her condition.

## 3. Discussion

We reported an unusual case of probable human coinfection of *Leptospira* and *Rickettsia*, and although it unfortunately involved preventable situations and a fatal outcome in a pediatric patient, it may allow us to analyze how important it is to develop quality initial anamnesis in order to visualize animal and human health as profoundly interconnected, as proposed by the One Health approach. The One Health approach involves multiple dimensions of health, aiming at the well-being of humans, animals, and the environment while considering relevant aspects, such as the access to safe water and waste management [15]. It is particularly necessary in regions where poverty may serve as a conditioning factor and a limitation to access to quality health services.

Clinical diagnosis is based on medical examination supported by laboratory tests and conducted with related medical history with consideration of the epidemiological and environmental context. While photophobia, gastrointestinal manifestations, pulmonary and renal involvement along with aseptic meningitis are consistent with *Leptospira*, the dermatologic manifestations, hepatomegaly, lymph node enlargement, and neurological manifestations are more frequently observed in rickettsiosis cases in the studied region [16,17]. In the case presented here, tests for these infective agents were not simultaneously developed at the public institution. Future considerations should explore the cost efficiency of simultaneous testing in severe cases in which treatment needs to be provided in an accurate way as soon as possible along with active surveillance.

Although initial clinical manifestations consisted mainly of signs and symptoms of unspecific acute, severe infections (e.g., fever, malaise, lymph node enlargement, hepatomegaly, and gastrointestinal manifestations), photophobia and progression to renal failure had previously been reported in severe leptospiral infections, while dermatologic manifestations and neurological impairment have been evidenced in fatal pediatric cases due to *Rickettsia* in the studied region [3,4,5,6,18]. Additionally, our patient presented with positive Kernig’s and Brudzinski’s signs consistent with leptospiral meningitis, which has been reported in approximately one quarter of cases among younger patients [9].

Human rabies transmitted by dogs was eradicated in Mexico as declared by the World Health Organization in 2019. With the latest cases followed in 2005 and confirmed in 2006, this public health goal was achieved by implementing vaccination campaigns for dogs since the 1990s with the coverage of more than 80%. Thus, rabies infection was unlikely in the studied patient [19].

Therapy for *Rickettsia* should be initiated within five days after observation of symptoms consistent with the signs and the epidemiological context. Penicillin, which was administered to the patient before hospitalization, if used in correct dosage, could have been useful against *Leptospira*, but doxycycline is commonly considered the most effective antibiotic against *Rickettsia* and has also proven to be useful against *Leptospira* [4,20]. Unfortunately, in the presented case, treatment with doxycycline had been delayed until the drug was available, by which time the patient had already had severe nonrefractory damage. Distribution of doxycycline has improved over the Mexican territory, but shortages are still common in the public health system. We believe that given the potential severity of *Rickettsia* infections in humans, this antibiotic should always be considered in the basic drug stock of both rural clinics and hospitals, particularly in southeast regions such as Yucatan, but also in the northern border region, including Sonora, Chihuahua, and Coahuila [21].

With the communication of this unusual yet regrettable case, we want to invite the scientific community from all disciplines to reflect on the possible different approaches for the study of these neglected pathogens in disadvantaged areas and to develop strategies to reduce their impact on the environment, animals, and humans.

The present case report has limitations that mainly derive from the lack of initial diagnostic tests and missing information on previous physical examinations at rural clinics, which could be due to a lack of resources, clinical suspicion, or both. Therefore, the interpretation of the present communication should be cautious. Nevertheless, the evidence of the medical attention, diagnostic procedures, treatments, and anamnesis at the capital city general hospital was extensive.

## 4. Conclusions

This case report exemplifies that a probable coinfection of *Rickettsia* and *Leptospira* may generate diverse signs and symptoms in a single patient and that these pathogens should be considered in endemic regions (a) if epidemiological association with a confirmed family member or infected domestic animals is found; (b) when the household environment and hygienic conditions are compatible with the transmission context of both pathogens; and (c) when signs and symptoms cannot be fully explained by a single pathogen and the epidemiological and clinical context suggest more than one possible causal agent, as in this case of *Rickettsia* and *Leptospira*. In such cases, simultaneous testing should be carried out to provide timely diagnosis and treatment; ultimately, testing should be extended to family members and involved animals in order to establish adequate preventive measures.

## Data Availability

No new data were created or analyzed in this study. Data sharing is not applicable to this article.
